# A nonsense mutation of *CRYGC* associated with autosomal dominant congenital nuclear cataracts and microcornea in a Chinese pedigree

**Published:** 2012-07-11

**Authors:** Yuanyuan Guo, Dongmei Su, Qian Li, Zhenfei Yang, Zicheng Ma, Xu Ma, Siquan Zhu

**Affiliations:** 1 Beijing Tongren Eye Center, Beijing Tongren Hospital, Capital Medical University, Beijing Ophthalmology & Visual Sciences Key Lab, Beijing, China; 2 National Research Institute for Family Planning, Beijing, China; 3 Peking Union Medical College, Beijing, China; 4 World Health Organization Collaborating Center for Research in Human Reproduction, Beijing, China

## Abstract

**Purpose:**

To report the identification of a nonsense mutation in γC-crystallin (*CRYGC*) associated with autosomal dominant congenital nuclear cataracts and microcornea in a Chinese family.

**Methods:**

We investigated four generations of a Chinese family six of whose members were affected by nuclear cataracts and microcornea. The genomic DNA was extracted from peripheral blood leukocytes. All reported nuclear cataract-related candidate genes were screened for causative mutations by direct DNA sequencing. The effects of amino acid changes on the structure and function of proteins were predicted by bioinformatics analysis.

**Results:**

All affected individuals in this family exhibited nuclear cataracts and microcornea. Direct sequencing of the candidate gene cluster showed a c.471G>A transition in exon 3 of *CRYGC*, which co-segregated according to family members with cataracts, and was not observed in 100 normal controls. This single nucleotide change was predicted to introduce a translation stop codon at tryptophan 157 (W157X). Bioinformatics analysis showed that the mutation was predicted to affect the function and secondary structure of the CRYGC protein.

**Conclusions:**

This study identified a disease-causing mutation c.471G>A in *CRYGC* in a Chinese family with cataracts, expanding the mutation spectrum of *CRYGC* causing congenital cataracts.

## Introduction

Congenital cataracts are one of the major causes of blindness and amblyopia in children. The prevalence of congenital cataracts is estimated to vary from 0.6 to 6 cases per 10,000 live births [1]. Hereditary factors account for approximately one quarter to one third of congenital cataracts [2]. Cataracts may be isolated, may be associated with other ocular anomalies, or may be part of multisystem genetic disorders, accounting for approximately 70%, 15%, and 15% of cases, respectively [3]. Inherited non-syndromic cataracts frequently exhibit autosomal dominant traits.

With the development of molecular biology techniques, more and more genes related to congenital cataracts have been mapped. So far, more than 40 loci have been mapped in isolated or primary congenital cataracts [4-6]. Several genes are highly expressed in the lens and have been associated with nuclear cataracts. They can be considered candidate genes for hereditary nuclear cataracts, and include αA-crystallin *(CRYAA*), βA1-crystallin (*CRYBA1*), βB1-crystallin (*CRYBB1*), βB2-crystallin (*CRYBB2*), γC-crystallin (*CRYGC*), γD-crystallin (*CRYGD*), connexin 46 (*CX46*), connexin 50 (*CX50*), and major intrinsic protein (*MIP*) [4,7,8]. Mutations in the *CRYG* gene cluster, located on 2q33–35, are the most frequent cause of autosomal dominant congenital cataracts.

In this study we used a functional candidate approach and investigated a Chinese family with autosomal dominant congenital nuclear cataract and microcornea. We detected a nonsense mutation in *CRYGC* that co-segregated with the disease in this family.

## Methods

### Clinical evaluation and DNA specimens

Four generations of a family with congenital nuclear cataracts and microcornea was recruited at Beijing Tongren Hospital, Capital Medical University, Beijing, China. Informed consent was obtained from all participants, consistent with the Declaration of Helsinki. Affected status was determined by a medical history or ophthalmologic examination, which included visual acuity, slit lamp examination, ultrasonography, intraocular pressure measurement, and fundus examination with dilated pupils. Phenotypes were documented using slit lamp photography. A total of 100 healthy normal controls were involved in the study. They were given complete ophthalmologic examinations identical to those of the cataract family participants, and did not have eye diseases except mild myopia and senile cataracts. Peripheral venous blood was collected and genomic DNA was extracted from blood leukocytes using a QIAamp DNA kit (Qiagen, Vlencia, CA).

### Mutation analysis

Nine candidate genes, including *CRYAA* (GenBank NM_000394.2), *CRYBA1* (GenBank NM_005208.4), *CRYBB1* (GenBank NM_001887.3), *CRYBB2* (GenBank NM_000496.2), *CRYGC* (GenBank NM_020989.3), *CRYGD* (GenBank NM_006891.3), *CX46* (GenBank NM_021954.3), *CX50* (GenBank NM_005267.4), and *MIP* (GenBank NM_012064.3), are highly expressed in the lens and have been associated with nuclear cataracts. These genes can be considered as candidate genes for hereditary nuclear cataracts [4,7,8]. Mutation screening was performed in these candidate genes. All coding exons and splice sites of the candidate genes were amplified by polymerase chain reactions (PCR) using the previously published primer sequences listed in Table 1 [8]. PCR products obtained from the proband and one unaffected member were sequenced on an ABI 3730 Automated Sequencer (PE Biosystems, Foster City, CA). The sequencing results were analyzed using Chromas 2.33 and compared with the reference sequence in the NCBI database. The samples from all available family members and 100 ethnically matched controls were directly sequenced to confirm the mutation identified in *CRYGC*.

**Table 1:** Polymerase chain reaction primers and product sizes.

**Gene**	**Forward primers (5′→3′)**	**Reverse primers (5′→3′)**	**Product size (bp)**
CRYAA-1	AGCAGCCTTCTTCATGAGC	CAAGACCAGAGTCCATCG	441
CRYAA-2	GGCAGGTGACCGAAGCATC	GAAGGCATGGTGCAGGTG	338
CRYAA-3	GCAGCTTCTCTGGCATGG	GGGAAGCAAAGGAAGACAGA	376
CRYBA1–1	GGCAGAGGGAGAGCAGAGTG	CACTAGGCAGGAGAACTGGG	207
CRYBA1–2	AGTGAGCAGCAGAGCCAGAA	GGTCAGTCACTGCCTTATGG	293
CRYBA1–3	AAGCACAGAGTCAGACTGAAGT	CCCCTGTCTGAAGGGACCTG	269
CRYBA1–4	GTACAGCTCTACTGGGATTG	ACTGATGATAAATAGCATGAACG	358
CRYBA1–5	GAATGATAGCCATAGCACTAG	TACCGATACGTATGAAATCTGA	291
CRYBA1–6	CATCTCATACCATTGTGTTGAG	GCAAGGTCTCATGCTTGAGG	295
CRYBB1–1	CCCTGGCTGGGGTTGTTGA	TGCCTATCTGCCTGTCTGTTTCTC	610
CRYBB1–2	TAGCGGGGTAATGGAGGGTG	AGGATAAGAGTCTGGGGAGGTGG	664
CRYBB1–3	CCTGCACTGCTGGCTTTTATTTA	TCTCCAGAGCCCAGAACCATG	475
CRYBB1–4	CCAACTCCAAGGAAACAGGCATA	CCTCCCTACCCACCATCATCTC	491
CRYBB1–5	TAGACAGCAGTGGTCCCTGGAGA	AGCACTGGGAGACTGTGGAAGG	416
CRYBB1–6	CCTAGAAAAGGAAACCGAGGCC	AGCGAGGAAGTCACATCCCAGTA	551
CRYBB2–1	GTTTGGGGCCAGAGGGGAGTGGT	TGGGCTGGGGAGGGACTTTCAGT	355
CRYBB2–2	CCTTCAGCATCCTTTGGGTTCTCT	GCAGTTCTAAAAGCTTCATCAGTC	597
CRYBB2–3	GTAGCCAGGATTCTGCCATAGGAA	GTGCCCTCTGGAGCATTTCATAGT	360
CRYBB2–4	GGCCCCCTCACCCATACTCA	CTTCCCTCCTGCCTCAACCTAATC	514
CRYBB2–5	CTTACCCTTGGGAAGTGGCAATGG	TCAAAGACCCACAGCAGACAAGTT	430
CRYGC-1	TGCATAAAATCCCCTTACCG	CCTCCCTGTAACCCACATTG	556
CRYGC-2	TGGTTGGACAAATTCTGGAAG	CCCACCCCATTCACTTCTTA	491
CRYGD-1	CAGCAGCCCTCCTGCTAT	GGGTCCTGACTTGAGGATGT	484
CRYGD-2	GCTTTTCTTCTCTTTTTATTTCTGG	AAGAAAGACACAAGCAAATCAGT	395
CX46–1	CGGTGTTCATGAGCATTTTC	CTCTTCAGCTGCTCCTCCTC	396
CX46–2	GAGGAGGAGCAGCTGAAGAG	AGCGGTGTGCGCATAGTAG	498
CX46–3	TCGGGTTCCCACCCTACTAT	TATCTGCTGGTGGGAAGTGC	596
CX50–1	CCGCGTTAGCAAAAACAGAT	CCTCCATGCGGACGTAGT	399
CX50–2	GCAGATCATCTTCGTCTCCA	GGCCACAGACAACATGAACA	400
CX50–3	CCACGGAGAAAACCATCTTC	GAGCGTAGGAAGGCAGTGTC	378
CX50–4	TCGAGGAGAAGATCAGCACA	GGCTGCTGGCTTTGCTTAG	375
MIP-1	GTGAAGGGGTTAAGAGGC	GGAGTCAGGGCAATAGAG	561
MIP-2	CGGGGAAGTCTTGAGGAG	CACGCAGAAGGAAAGCAG	847
MIP-3	CCACTAAGGTGGCTGGAA	CTCATGCCCCAAAACTCA	561

### Bioinformatics analysis

The multiple-sequence alignment of the amino acid sequence in CRYGC from several different species was analyzed by the CLC Free Workbench 4.5.1 software (CLC bio, Aarhus, Denmark). Both mutant and wild-type versions of the protein structure were predicted and analyzed by the SWISS-MODEL online tool [9-11].

## Results

### Clinical findings

**Figure 1:**
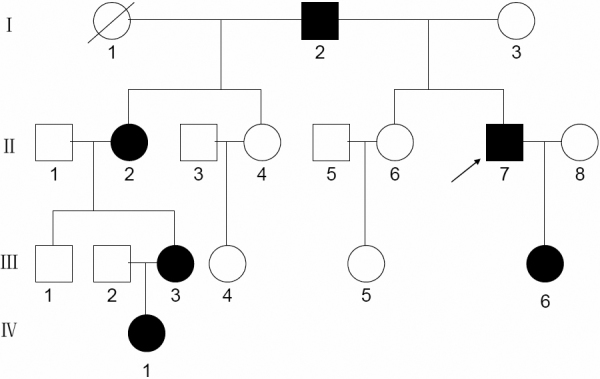
Pedigree of four generations of a family with autosomal dominant cataracts. Squares and circles indicate males and females, respectively. Black and white symbols denote affected and unaffected individual, respectively. The black arrow indicates the proband.

**Figure 2:**
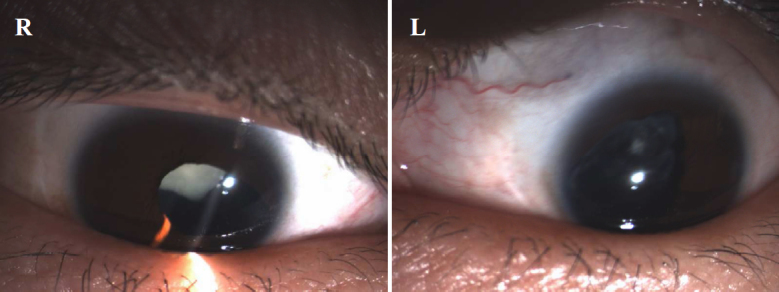
Slit lamp photographs of the proband (II:7). The nuclear opatities of the lens were involved the embryonal, fetal, and infantile nuclei. The patient underwent iridectomy on both eyes at the age of 8 years. The left eye underwent phacoemulsification and intraocular lens implantation at the age of 38 years. The diameter of bilateral corneas is approximately 9 mm.

We identified isolated bilateral autosomal dominant nuclear cataracts and microcornea in a four-generation Chinese family (Figure 1). The nuclear opacities were located in the embryonic, fetal, and infantile nuclei. Bilateral nuclear cataracts and microcornea with a diameter of approximately 9 mm were consistent in all of the affected individuals. According to patients' personal reasons,they underwent examinations and surgery at different ages. The proband (II:7) was diagnosed with bilateral nuclear cataract and microcornea at the age of 8. He underwent iridectomy on both eyes at the age of 8. His left eye underwent phacoemulsification and intraocular lens implantation at the age of 38. His best corrected visual acuity in the right and left eyes, was 0.08 and FC/30cm, respectively (Figure 2). Individual I:2 underwent the examinations at the age of 68. Individuals III:6 and IV:1 were first diagnosed with bilateral nuclear cataracts and microcornea respectively at 6 months-old and 1 year-old. Individual II:2 was diagnosed at 13 years of age. Individual III:3 had cataract extraction performed at 6 years of age. According to the examinations and medical records, all patients in this family showed nuclear cataracts and microcornea. The intraocular pressure of the patients was normal. Nystagmus and amblyopia were observed in all the patients. There was no history of other related systemic abnormalities in this family.

**Figure 3:**
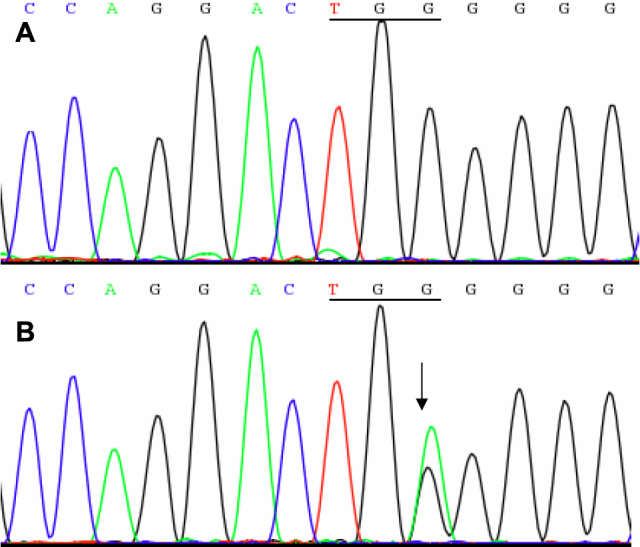
Forward sequence analysis of *CRYGC*. **A**: DNA sequence chromatograms of an unaffected member (II:8) shows TGG at codon 157. **B**: DNA sequence chromatograms of an affected member (II:7) shows a G>A transition that changed Trp to a stop codon (TGA).

Mutation analysis: Direct sequencing of the coding regions of the nine candidate genes was performed and a transition of G>A was identified at c.471 in exon 3 of *CRYGC* (Figure 3). This mutation resulted in the substitution of a phylogenetically conserved tryptophan residue to a stop codon (W157X). The transition c.471G>A was not found in any of the unaffected family members or in the 100 controls. No other mutations were found except for a few nonpathogenic single nucleotide polymorphisms.

### Bioinformatics analysis

**Figure 4:**
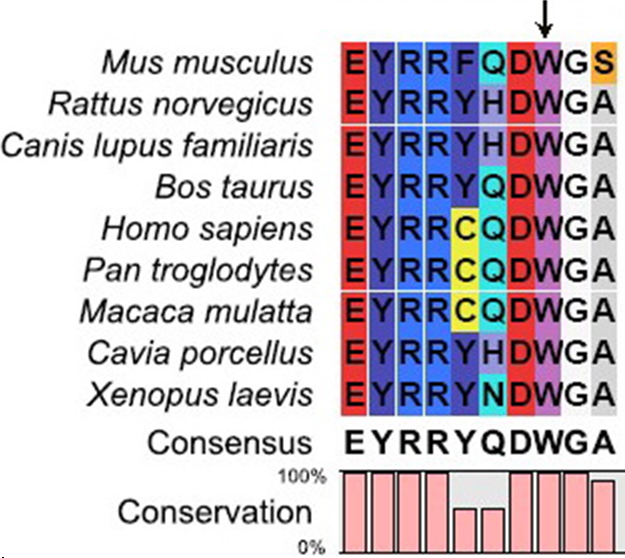
A multiple-sequence alignment of the amino acid sequence in CRYGC from different species. The alignment data indicates that the Trp at the W157X mutated position is highly conserved in different species (indicated by an arrow).

**Figure 5:**
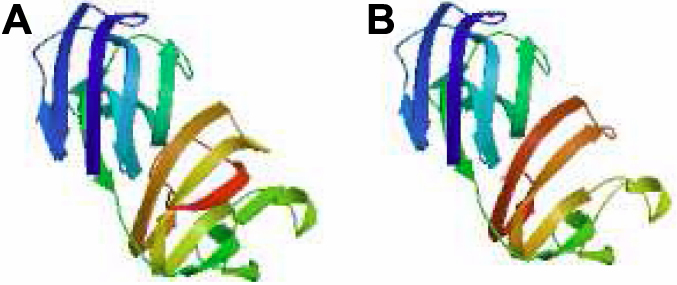
Protein models of the wild-type and mutant γC-crystallins. **A**: A structural model of the wild-type γC-crystallin is displayed. **B**: A structural alteration of the mutant γC-crystallin is displayed. Eighteen amino acids are truncated from the COOH-terminus of γC-crystallin as result of c.471G>A mutation.

The mutant position that the Trp occupies at the W157X is highly conserved in different species (Figure 4). There were 18 amino acids less than the wild-type CRYGC. The fourth Greek key motif at the COOH-terminus was found to be partially absent in the three-dimensional structural model of the mutant CRYGC (Figure 5).

## Discussion

In this study, we report a nonsense mutation (c.471G>A) in exon 3 of *CRYGC* resulting in autosomal dominant congenital nuclear cataracts and microcornea in a four-generation Chinese family. It co-segregated within the family and did not occur in the unaffected family members or in the 100 ethnically matched controls.

The mammalian lens crystallins are essential in maintaining lens transparency and divided into α-, β-, and γ-crystallins families. The γ-crystallins are characterized as monomers and have a low molecular mass of 21 kDa. The γC-crystallins are the most common type of γ-crystallins and expressed in young human lens. Furthermore, the γC-crystallins are primarily localized in the lens nucleus [12,13]. They are considered a member of a β/γ-crystallin superfamily which has the Greek key motif (GKM). The protein regions in γ-crystallins include four Greek key motifs, a connecting peptide, NH_2_-terminal extensions, and COOH-terminal extensions. GKM1 and GKM2 are located in the NH_2_-terminal domain and GKM3 and GKM4 are located in the COOH-terminal domain. Each Greek key motif is composed of four antiparallel β-strands and the four Greek key motifs form four β-sheets [14-16]. The mutant domain W157X is located in GKM4.

The transition of G>A at c.471 in exon 3 of *CRYGC* is predicted to cause a premature stop codon. The nonsense-mediated mRNA decay (NMD) pathway is an effective mRNA surveillance system that detects and degrades mRNA containing premature termination codons (PTC) and protects cells from the potentially deleterious effects of truncated proteins. However, some PTC-mRNAs can escape the NMD [17-19]. The mutant transcripts of this nonsense mutation (c.471G>A, p.W157X) may escape the NMD pathway and the aberrant PTC-mRNAs are translated to abnormal proteins with potential dominant-negative effects in cells. The mutation identified here, p. W157X *CRYGC*, lies in a highly conserved amino acid located in the COOH-terminal domain at the end of the fourth Greek key motif. Trp at the W157X mutated position is highly conserved in different species by multiple-sequence alignment (Figure 4). The W157X mutation may damage an important function of this region in CRYGC. The mutation detected in our present study, c.471G>A, creates a premature stop codon (W157X) and results in an in-frame stop codon at nucleotide 73 of exon 3 that may cause the truncation of 18 amino acids from the COOH-terminus of γC-crystallin. This alteration correspondingly affects the formation of the fourth Greek-key motif in γC-crystallin, and then disrupts the highly symmetric structure of γC-crystallin. The W157X mutation is predicted to change the folding properties of γC-crystallin and may affect its interactions with other crystallins. A previous W157X mutant investigation found that the loss of the COOH-terminal 18 residues led to the exposure of several apolar residues (such as Leu57, Ile112, Ile121, Trp131, and Leu133) buried by the COOH-terminal region in the wild-type. The results included the markedly decreased solubility of the molecule and the formation of substantial intermolecular aggregates in a variety of cells, notably in human lens epithelial cells. Such aggregates would be expected to generate light-scattering particles, compromising the transparency of the cells and their assemblies [20]. All in all, the truncated γC-crystallin in the present study not only disrupts the structure of the Greek key motif but may also alter the interactions between γC-crystallin and other crystallins.

Mutations in the human *CRYGC* gene causing congenital cataracts have been identified in seven families, as listed in Table 2 [21-27]. In addition to the c.471G>A mutation, the other phenotypes described in those studies showed Coppock-like cataracts, zonular pulverulent cataracts, lamellar cataracts, nuclear cataracts, and nuclear and microcornea cataracts. The W157X mutation was not only observed in our family but also in another previously described family with nuclear cataracts and microcornea [26], indicating that W157X is the actual disease-causing mutation and should no longer be considered a single nucleotide polymorphism. Although the results of the two papers revealed the same W157X change of the CRYGC protein, the G>A transition sites were different. The site of G>A transition in the previous W157X report was c.470G>A (TGG→TAG) in *CRYGC*. However, in our paper, the site of G>A transition was c.471G>A (TGG→TGA) in *CRYGC* (Table 2). The previous pedigree is a four-generation Chinese family that resides in a relatively isolated region of northern China. The two families have different pedigrees. The c.471G>A transition in *CRYGC* is the first reported. All patients in this family showed nuclear cataracts and microcornea, and the cornea diameter was approximately 9 mm. The previous W157X report was a c.470G>A transition and the clinical phenotype also was nuclear cataracts and microcornea. The phenotypes of these two mutations were similar. The microcornea is one of the most common abnormalities associated with congenital cataracts, further emphasizing the interdependence of the lens and cornea in development and metabolism [4]. This study further confirms that the region of mutant W157X in *CRYGC* is a critical locus in cataractogenesis in humans.

**Table 2:** Human *CRYGC* mutations associated with congenital cataract.

**Mutation**	**Amino acid change**	**Protein domains**	**Cataract phenotype**	**Reference**
c.13A>C	T5P	GKM1	Coppock-like	[21]
c.123–128insGCGGC	52 new aa	GKM2	Zonular pulverulent	[22]
c.502C>T	R168W	GKM4	Lamellar	[23]
c.502C>T	R168W	GKM4	Nuclear	[24]
c.327C>A	C109X	GKM3	Nuclear	[25]
c.470G>A	W157X	GKM4	Nuclear + Microcornea	[26]
c.385G>T	G129C	GKM4	Nuclear	[27]
c.471G>A	W157X	GKM4	Nuclear + Microcornea	Present study

GKM=Greek key motif.

In conclusion, the present study has identified a nonsense mutation (c.471G>A, p.W157X) in *CRYGC* associated with autosomal dominant congenital nuclear cataracts and microcornea in a Chinese family. The predicted change of the protein structure may disrupt lens biochemistry and physiology early in development. The possible mechanism of this mutation will require further investigation.
